# Maternal health care utilization and the obstetric outcomes of undocumented women in Finland – a retrospective register-based study

**DOI:** 10.1186/s12884-021-03642-7

**Published:** 2021-03-06

**Authors:** Janita Tasa, Ville Holmberg, Susanna Sainio, Päivi Kankkunen, Katri Vehviläinen-Julkunen

**Affiliations:** 1 Department of Nursing Science, University of Eastern Finland, Kuopio, Finland; 2 Inflammation Center, Infectious Diseases, Helsinki University Hospital, Helsinki, Finland; 3 Clinicum, Department of Medicine, University of Helsinki, Helsinki, Finland; 4 Finnish Red Cross Blood Service, Helsinki, Finland; 5 Clinical Development, Education and Research Centre, Kuopio University Hospital, Kuopio, Finland

**Keywords:** Undocumented, Migrant, Prenatal care, Pregnancy, Childbirth, Access to health care, Register-based study

## Abstract

**Background:**

Undocumented pregnant women constitute a vulnerable group of people who lack equal access to pregnancy care. Previous research has shown that undocumented migrants encounter difficulties in accessing health services, the onset of prenatal care is delayed, and women have an increased risk for infectious diseases. The aim of this study was to describe the use of maternal health care services and the obstetric outcomes of undocumented women in Helsinki, capital city of Finland, in addition to comparing the results with all pregnant women in Finland.

**Methods:**

The study was a retrospective register-based study consisting of data collected between 2014 to 2018 from the electronic medical records of the public maternity clinic and maternity hospital in Helsinki, Finland. The study population consists of 62 individual pregnancies of undocumented women. The results of the study were compared with national data on parturients and deliveries (*N* = 47,274 women) and with prenatal screening tests for infectious diseases (*N* = 51,447 [HIV, HBV], N = 51,446 [syphilis]).

**Results:**

The majority (91%) of the undocumented women attended public prenatal care. However, four women received no prenatal care and three women were denied access to care. Undocumented women entered prenatal care later and had fewer visits compared with all pregnant women. The majority (71%) of the undocumented women received inadequate prenatal care as the number of visits was less than eight. Of the study population, 5% (3/59) tested positive for HIV, 3% (2/59) for HBV, and 2% (1/57) for syphilis. The prevalence of HIV (*p*-value < 0.001) and HBV (p-value = 0.007) was significantly higher amongst undocumented women compared with all pregnant women.

**Conclusions:**

Undocumented women entered prenatal care later than recommended. Most women received inadequate prenatal care and some of them did not receive prenatal care at all. The prevalence of infectious diseases was significantly higher and the coverage of prenatal screenings deficient amongst undocumented pregnant women.

## Background

Every woman should be entitled to special care and assistance during pregnancy, childbirth, and the postnatal period [1–3]. Undocumented pregnant women constitute a particularly vulnerable group of people [4–9] who lack equal access to quality pregnancy care [9]. With continuous prenatal care throughout pregnancy, it is possible to prevent pregnancy complications and arrange proper medical care and guidance when needed [3].

An undocumented migrant is a person who does not have a valid permit to reside in a country. A person may end up as undocumented, for example, as a result of an expired visa or resident permit, a rejected asylum claim, or while waiting for a resident permit [10, 11]. In addition, in Finland, those EU citizens, who do not have a health insurance to cover the costs of medical care, are considered undocumented migrants [10].

Previous research has shown that undocumented migrants encounter great difficulties in accessing health services [8, 11, 12]. Undocumented pregnant women use less health care services compared with other women in the same country [6, 7, 9, 13–15] and the onset of prenatal care is often delayed [4, 5, 9, 13, 16–18].

Previous research has also found that undocumented women have an increased risk for sexually transmitted diseases (STD) [4, 17, 19–21] and majority of them are not adequately screened [4, 17]. According to guidelines of the European Center for Diseases Prevention and Control (ECDC), all migrants from high-prevalence countries (> 1%) should be tested for human immunodeficiency virus (HIV), and pregnant migrants are a priority group for screening. All HIV-positives should immediately be linked to HIV care and treatment programs in accordance with national clinical guidelines. Hepatitis B (HBV) screening of all pregnant migrants is recommended irrespective of the prevalence in the country of origin [22].

In Finland, according to the national legislation, undocumented pregnant women do not have access to preventive maternal health care services, as they are only provided to Finnish residents. However, urgent medical care is provided to everyone regardless of the nationality or the place of residence of the person. Thereby, undocumented women have access to medical care only in the case of pregnancy complications or during childbirth [23]. However, municipalities are entitled to provide wider access to health care for undocumented migrants [24]. Some municipalities, including Helsinki, have decided to provide undocumented pregnant women with access to same public maternal health care services with same expenses as for residents. Basic health care services for undocumented migrants are also provided by health care professionals at voluntarily run ‘Global Clinics’ in five different cities in Finland, one clinic operating in Helsinki [25].

Since there is evidence that the number of undocumented migrants in Finland has grown in recent years due to an increase in migration [26] and an increase in the number of rejected asylum applications [27], it is highly relevant to recognize and address the health needs of this vulnerable minority group [6]. The present study aimed at identifying a) the characteristics of undocumented pregnant women, b) the utilization of maternal health care services in Helsinki, and c) the obstetric outcomes of undocumented women in addition to comparing the outcomes with all pregnant women in Finland.

## Methods

### Study setting

The study was conducted in Helsinki, the capital of Finland, a country with a population of 5.5 million [28]. Of the approximately 643,000 residents of Helsinki, 10% are foreign nationals [29]. In Finland, the estimated number of undocumented migrants is 3000–4000 [10, 27], of whom 100–1000 live in Helsinki [28]. In all of Europe, in 2017, the estimated number of undocumented migrants was 3.9–4.8 million [30].

Since 2013, undocumented pregnant women have had access to maternal health care services in Helsinki [31, 32]. In Finland, women are recommended to have their first prenatal visit during the first pregnancy trimester at gestation weeks 8–10 and to attend eight to ten prenatal care visits during pregnancy [33]. Prenatal care at the public maternity clinic is free of charge [32]. In this study, having fewer than eight prenatal care visits was considered as inadequate prenatal care and having less than four prenatal care visits was considered as clearly inadequate prenatal care.

### Study population

The study population consists of all undocumented pregnant women (*n* = 60) that were identified in the electronic medical records of the public maternity clinics in Helsinki between June 2014 to May 2018. Two undocumented women within the study population were pregnant twice during the study period. Thus, the study covers altogether 62 pregnancies (*N* = 62). The results of the study were compared with national data on parturients, deliveries, and newborns in 2018 (*N* = 47,274 women) and with prenatal screening tests for infectious diseases in 2017 (*N* = 51,447 [HIV, HBV], N = 51,446 [syphilis]). The report on the perinatal statistics [34] is publicly available and the data on infectious diseases [35] was obtained from the Finnish Institute for Health and Welfare.

### Study design and data

This study was a descriptive cross-sectional study using retrospective register-based data. The study consists of data collected from two distinct electronic medical record systems: 1) *Pegasos* – an electronic medical record system used in the public maternity clinics of primary health care in Helsinki and 2) *Obstetrix* – an electronic medical record system used in the public maternity hospitals of specialized medical care in Helsinki and Uusimaa Hospital District. Since 2014, maternity clinics in Helsinki have used a unique statistical code enabling the identification of undocumented pregnant women in Pegasos. Linking data from separate electronic medical records is possible by using identifying information.

The data consists of primary outcome variables: *prenatal care utilization* (the provider of prenatal care, timing of first prenatal visit, number of all prenatal visits, reason for ending prenatal care, number of missed prenatal visits, and the use of an interpreter) and *screening of infectious diseases* (HIV, hepatitis B/C, syphilis, chlamydia trachomatis, methicillin-resistant *Staphylococcus aureus* [MRSA], extended spectrum beta-lactamases [ESBL], and group B streptococcus [GBS]). In addition, secondary outcome variables: *characteristics of the study population* (maternal age, parity, smoking habit, use of alcohol, marital status, country of origin, reason for migration, and reason for being in an undocumented situation) and *birth outcomes* (gestational age at birth, birth outcome, birth weight of the newborn, Apgar score, and neonatal admission).

Individual variables such as first prenatal visit, number of prenatal visits, vacuum extraction, caesarean section, birth weight of the newborn, neonatal admission, screening and positive test result of HIV, HBV, and syphilis were compared between the study population and the comparison group.

### Statistical analyses

The statistical analyses were conducted using IBM SPSS statistics 24 for Mac and Stata 16.1. The data were entered into SPSS anonymously: women in the study population were numbered sequentially (1–62). Descriptive statistics were undertaken presenting frequencies, percentages, mean, standard deviation, minimum, and maximum values of the variables. Individual variables (maternal age, country of origin, timing of first prenatal visit, and number of prenatal visits) were categorized before conducting statistical calculations. In some cases, individual variables lacked values or the information available was unclear. These were entered into SPSS as ‘missing information’ and excluded from the analyses.

Binominal Exact test was used to calculate the 95% confidence intervals of the prevalence of HIV, HBV, syphilis and the proportion of vacuum extraction, caesarian section, and neonatal admission of undocumented pregnant women and all pregnant women. *P*-values for differences between the above-mentioned variables in the two groups were calculated using Fisher’s Exact test. For continuous variables (first prenatal visit, number of prenatal visits, and birth weight of the newborn) two-sample t-test was used to calculate the 95% confidence intervals and *p*-values between the two groups.

## Results

### Characteristics of the study population

The main characteristics of the study population are presented in Table 1. More than half (56%; 35/62) of the women were primiparous and their mean age was 28 years (SD ± 4.95, range 17–42 years). The majority (85%; 45/53) of women were married or cohabiting.

**Table 1:** Characteristics of the study population (*N* = 62)

	n (%)
**Age (years)**	
17–24	13 (21)
25–29	28 (45)
30–34	13 (21)
35–42	8 (13)
**Parity**	
0	35 (56)
1–2	22 (35)
3–5	5 (9)
**Smoking** ^a**,**b^	
No	48 (83)
Continued throughout pregnancy	6 (10)
Quit smoking when pregnant	4 (7)
**Alcohol use** ^a**,**b^	
No	54 (96)
Yes	2 (4)
**Marital status** ^a**,**b^	
Married/cohabiting	45 (85)
Relationship, not living together	5 (9)
Unmarried	3 (6)
**Country of origin** ^b^	
Eastern Europe and Russia	21 (36)
Sub-Saharan Africa	12 (20)
Asia	11 (18)
Middle East	9 (15)
North Africa	5 (8)
South America	2 (3)
**Reason for migration** ^b^	
Family	35 (58)
Work	11 (18)
Study	4 (7)
Other	10 (17)
**Reason for being in an undocumented situation** ^a**,**b^	
Applying for resident permit	33 (57)
EU citizen	13 (23)
Illegally in the country	7 (12)
Student	2 (3)
Other	3 (5)

^a^Missing data excluded

^b^ *n* = 60. Two women were pregnant twice. Duplicate removed

Altogether, the women came from 25 different countries – the majority of them originating from Eastern Europe and Russia (36%; 21/60), Sub-Saharan Africa (20%; 12/60), Asia (18%; 11/60), and the Middle East (15%; 9/60). Family ties (58%; 35/60) was clearly the main reason for migration. Women were in an undocumented situation for different reasons. More than half (57%; 33/58) of the women were applying for residency, one-fifth (23%; 13/58) were EU citizens, and one-tenth (12%; 7/58) were illegally in the country (Table 1.)

### Access to prenatal care

The majority of the women (91%; 56/62) attended public prenatal care. Only four women (6%; 4/62) had no prenatal visits before delivery. More than one-tenth (13%; 8/62) of the undocumented women had also visited Global Clinic at least once. Three women (5%; 3/60) were denied access or continuation of prenatal care by a health care professional (Table 2.)

**Table 2:** Prenatal care utilization (N = 62)

	n (%)
**Prenatal care provider**	
Public maternity clinic	50 (81)
Voluntary clinic for undocumented migrants, Global Clinic (GC)	2 (3)
Partly a maternity clinic and GC	6 (10)
No prenatal care	4 (6)
**Prenatal visit before arrival to Finland**^a^	
Yes	10 (19)
No	44 (81)
**Reason for ending prenatal care** ^a,b^	
Yes	21 (35)
Moved out of Finland	9 (15)
Prenatal care continues at a private clinic or in another city	5 (8)
Care denied	3 (5)
Unknown reason	4 (7)
No	39 (65)
**First prenatal visit** ^a^	
1. trimester	22 (39)
2. trimester	19 (34)
3. trimester	15 (27)
**Number of prenatal visits**	
0–3	25 (40)
4–7	19 (31)
8–10	12 (19)
≥ 11	6 (10)
**Number of missed prenatal visits** ^a^	
0	38 (70)
1	7 (13)
2	5 (9)
3	4 (8)
**Use of interpreter during prenatal visit** ^a^	
Family member	15 (25)
Professional interpreter ^c^	15 (25)
Both family member and professional interpreter	8 (13)
Interpreted not needed	19 (32)
Interpreter not used	3 (5)

^a^ Missing data excluded

^b^ Two of the study population women were still pregnant during the data collection

^c^ Including translation via phone

The onset of prenatal care was delayed – 61% (34/56) of the undocumented women attended their first prenatal visit during the second or third pregnancy trimester (Table 2). The mean gestation week at the first prenatal visit of undocumented women was 20.5 (SD ± 10.3, range 8.0–38.9), which was significantly later than compared with all pregnant women (*p* <  0.001). Undocumented women also attended prenatal care less frequently (p <  0.001) compared with all pregnant women, as the mean number of prenatal visits was 5.2 (SD ± 3.9, range 0–15) (Table 5).

The majority of undocumented women (71%; 44/62) received inadequate (< 8 visits) prenatal care and of these, 40% (25/62) clearly inadequate prenatal care (0–3 visits). Nonetheless, one-tenth (10%; 6/62) of the women received more care than was recommended (≥ 11 visits). Interpretation during prenatal visits was used in the majority (63%; 38/60) of the situations where it was needed. Though solely professional interpretation was used in one-fourth (25%; 15/60) of the prenatal visits (Table 2).

### Obstetric outcomes

#### Screening of infections

The results of this study showed that three (5%; 3/59) undocumented women tested positive for HIV (HIVAgAb), two (3%; 2/59) for HBV (HBsAg), and one (2%; 1/57) for syphilis (Treponema pallidum antibody). One woman tested positive simultaneously for HIV, HBV, and syphilis. The prevalence of HIV (*p*-value < 0.001) and HBV (p-value = 0.007) was significantly higher amongst undocumented woman compared with all pregnant women. Of the women, only 43% (26/60) were screened for hepatitis C (HCV) antibodies and all samples turned out negative (Table 3). In addition, screening tests for multidrug resistant bacteria showed that three (14%; 3/21) women were colonized with MRSA and two (10%; 2/21) women were diagnosed with ESBL infection. GBS test came out positive amongst 20% (9/44) of the women.

**Table 3:** The prevalence of infectious diseases between undocumented women (n = 60) and all pregnant women (*N* = 51,447) in 2017

	Undocumented women			All pregnant women		
	**Screening completed n (%)**	**Positives n**	**Prevalence % (95%CI)**	**Positives n**	**Prevalence % (95%CI)**	**p-value**^a^
HIV	59 (98)	3	5.1 (1.1–14.1)	42	0.08 (0.06–0.1)	< 0.001
HBV	59 (98)	2	3.4 (0.4–11.7)	110	0.2 (0.2–0.3)	0.007
Syphilis	57 (95)	1	1.8 (0.04–9.4)	43	0.08 (0.06–0.1)^b^	0.048
HCV	26 (43)	0	0 (−)	–	–	–
Chlamydia trachomatis^c^	17 (28)	1	5.9 (–)	–	–	–

Register data on all pregnant women who underwent infectious disease screening in 2017 [35]

Duplicate pregnancies excluded from the analysis

^a^ Fisher’s Exact test

^b^ N = 51,446

^c^ Not part of routine prenatal screening. Tested if symptoms occurred

#### Birth outcomes

The mean gestational age at birth was 40.1 weeks (SD ± 1.3). The majority of the undocumented women (87%; 41/47) delivered vaginally. Caesarean section was performed to 13% (6/47) of the women (Table 4). There was no statistically significant difference between the proportion of vacuum assisted deliveries or caesarian sections amongst undocumented women and all pregnant women (Table 5). There were no preterm deliveries (< 37.0), low birth weight babies (< 2500 g), nor stillbirths (Table 4).

**Table 4:** Birth outcomes of undocumented pregnant women (n = 47)

	**n (%)**	**Mean**	**SD**	**Min**	**Max**
**Gestational age at birth (gw)**		40.1	1.3	37.1	42.3
**Birth outcome**					
Spontaneous vaginal delivery	33 (70)				
Vacuum extraction	8 (17)				
Caesarean section	6 (13)				
**Still birth**	0 (0)				
**Birth weight (g)**		3438	380.6	2642	4125
**Apgar score at 5 min**^a^		9	0.9	6	10
**Umbilical cord pH**					
< 7.10	1 (2)				
7.10–7.20	14 (30)				
> 7.20	32 (68)				
**Neonatal admission**^b^	3 (6)				

^a^ Missing data excluded

^b^ Respiratory distress and hypoglycemia reasons for admission to neonatal intensive care unit

**Table 5:** Obstetric outcomes between undocumented women (*N* = 62) and all pregnant women (*N* = 47,274) in 2018

	**Undocumented women**		**All pregnant women**		
	**mean (95% CI)**		**mean (95% CI)**		***p*-value**^**a**^
**First prenatal visit** (gw)	20.5 (17.9–23.1)		9.5 (9.5–9.5)		< 0.001
**Number of prenatal visits**	5.2 (4.2–6.2)		13.4 (13.3–13.5)		< 0.001
**Birth weight of newborn** (g)	3438 (3341–3534)^c^		3499 (3494–3504)^d^		0.38
	**n**	**proportion % (95% CI)**	**n**	**proportion % (95% CI)**	***p*-value**^**b**^
**Vacuum extraction**	8^c^	17.0 (7.6–30.8)^c^	4598	9.7 (9.5–10.0)	0.13
**Caesarean section**	6^c^	12.8 (4.8–25.7)^c^	7899	16.7 (16.4–17.0)	0.56
**Neonatal admission**	3^c^	6.4 (1.3–17.5)^c^	5609^d^	11.7 (11.5–12.0)^d^	0.36

Register data on parturients, deliveries, and newborns in 2018 [34]

^a^Two sample t-test

^b^Fisher’s Exact test

^c^*n* = 47 (undocumented parturients and live born neonates)

^d^*n* = 47,777 (live born neonates)

## Discussion

This is the first study addressing the use of maternal health care services and obstetric outcomes by undocumented women in Finland. This study demonstrates three highly important issues: 1) undocumented women attended maternal health care services in a late stage of pregnancy, 2) the number of prenatal visits was inadequate, and 3) the prevalence of infectious diseases was significantly higher amongst undocumented women.

### Access to prenatal care

In this study, most undocumented women had their first prenatal visit as late as in the second or third pregnancy trimester which is later than the Finnish Institute for Health and Welfare recommends [33]. However, according to the electronic medical records, there was no information available on how long the undocumented women had resided in Finland before accessing their first prenatal visit. Undocumented women had their first prenatal visit even later compared with women in other European countries [4, 5, 9, 16–18]. In addition, undocumented women entered prenatal care on average ten weeks later compared with all pregnant women in Finland [34]. The onset of prenatal care of undocumented women in Helsinki was also delayed compared with the results of de Jonge et al. [5], Wolff et al. [16], and Paquier et al. [18] as their studies showed undocumented women entering prenatal care one to five weeks later in comparison to other women in the same country.

In addition to delayed prenatal care, the results of this study showed undocumented women receiving inadequate prenatal care. This is consistent with previous research [6, 7, 9, 13–15]. Moreover, the average number of prenatal visits of undocumented women was inadequate compared with the Finnish Institute for Health and Welfare’s recommendation of eight to ten visits [33], in addition to the World Health Organization’s recommendation of minimum of eight visits [3]. Undocumented women in Helsinki had eight prenatal visits less than the average of all pregnant women in Finland in 2018 [34]. This study showed that almost one-fifth of the undocumented women had visited a health care professional at least once before arrival to Finland. Nevertheless, we suggest that the undocumented women received inadequate prenatal care as there was no reliable information or documentation available of these visits.

An additional finding of concern is that four women in this study did not receive any prenatal care. Similar results have been found in Canada, where 7% [14], and in the Netherlands, where nearly one-fifth of the undocumented women received no prenatal care [4]. In addition, this study showed that three women were denied access or continuation of care by a health care professional. Previous research from Norway and Sweden, and a report from the Swedish Agency for Public Management have found similar tendencies [8, 36, 37].

As undocumented women have had access to public maternal health care in Helsinki already since 2013 [31], it is necessary to consider why women access these services in such a late stage of pregnancy. In this study, inadequate prenatal care can partially be explained by the late onset of prenatal care, women ending prenatal care, and women not showing up for booked appointments. Previous research also suggests that lack of information on the right to health services, fear [4, 7, 36], unfamiliar health care system, and poor financial situation [4, 11] may lead to under-utilization of health care services. It is also worth highlighting that language barriers may complicate the access to and quality of care [12], in addition to the delivery of maternity care [9]. In this research, solely professional interpretation was used only in one-fourth of the prenatal visits, which is less frequently compared with a recent study conducted in Sweden, where professional interpretation was used in 82% of the prenatal visits of asylum seeker and undocumented pregnant women [9]. Lastly, it needs to be considered that the lack of knowledge amongst health care professionals on the official guidelines on the legal rights of undocumented migrants may hinder the access to prenatal care [7, 8, 37].

The results of this study, amongst previous research [4, 9, 14, 17], underlines the importance of early access to maternal health care services for undocumented women. There is evidence to suggest that by expanding access to prenatal care, the utilization [15, 38] and the quality of care increased, and undocumented women were more likely to receive adequate prenatal care [38]. Furthermore, late access to health services may implicate higher financial costs as timely treatment in the primary health care setting has shown to be more cost-effective compared with urgent medical care [39, 40].

As the legal right to maternity health care is not sufficient to guarantee access to prenatal care [9], there is a need to develop diverse and innovative ways to reach undocumented women to increase access to care. Further, the importance of policy-makers and health care managers’ responsibility in ensuring that health care professionals receive sufficient training and knowledge on the rights and practices of care of undocumented pregnant women should be emphasized [8, 37].

### Screening of infections

There is only scarce research on the prevalence of infectious diseases amongst undocumented pregnant women. In this study, the prevalence of HIV and HBV was significantly higher amongst undocumented women compared with all pregnant women. The results are in line with previous research that has found the prevalence of infectious diseases higher amongst undocumented women compared with other migrant or native women in the same country [5, 17, 19, 20].

Interestingly, this study found that the prevalence of HIV amongst undocumented pregnant women in Helsinki was higher than what was found by Wolff et al. [16] (0.6%, *n* = 161) and Wendland et al. [17] (1.5%; *n* = 132). In contrast, the prevalence of HBV was less amongst undocumented women in this study than what Wendland et al. [17] had found (6.5%; *N* = 92). The prevalence of infections detected amongst the undocumented women in this study were high also compared with data from asylum seekers in Finland (HIV 0.3%; *n* = 45, HBV 1.6%; *n* = 275) [41] and asylum seekers in Italy (HIV 1.6%; *n* = 5, syphilis 0.7%; n = 2) [42]. Though, it is possible that these discrepant observations are due to different backgrounds in different populations or the small sample size in all the studies.

The Finnish Institute for Health and Welfare recommends screening for HIV, HBV, and syphilis in the early stage of pregnancy [33]. In addition, the official guidelines for prenatal and obstetric care issued by the Helsinki and Uusimaa hospital district recommends screening of HCV amongst undocumented women [43]. It is noteworthy that more than half of the women were not tested for HCV. The reason for low coverage of HCV testing may be explained by the fact that the guideline is not a national regulation, merely a local recommendation, and the fact that the health care professionals may not have been aware of the specific guideline. Restricting access to preventive and timely health care is not only unequal but may lead to adverse consequences for public health due to limited or late screening and treatment of communicable infections and mother-to-child transmissions [17].

### Strengths and limitations

This study was a register-based study conducted as a census study and the study population comprised exclusively of undocumented women. The study was conducted in Helsinki, the capital of Finland, where most undocumented migrants are assumed to reside. The heterogeneous study population represents well the population of undocumented pregnant women met in the maternity health care services and at Global Clinic in Helsinki. The reliability of this study is increased by the fact that Finnish health registers have been found to have good coverage, high validity, and data quality [44]. The accuracy of the data used is enhanced by the fact that many of the variables already existed as such in the electronic medical records of both primary (Pegasos) and specialized health care (Obstetrix).

The limitation of the study is its relatively small study population. However, it must be considered that the number of all undocumented migrants in all of Finland is estimated to be only 3000–4000 [10, 27]. The possibility that some undocumented women may have been left outside of the study cannot be excluded. This could be due to a failure to register them under the proper statistical code (primary health care) or due to the legal status of migrants not being registered at all (specialized health care).

## Conclusions

This study indicates that the onset of prenatal care of undocumented pregnant women was delayed. In addition, undocumented pregnant women received inadequate prenatal care and some of them did not receive prenatal care at all. The prevalence of HIV and HBV was significantly higher amongst undocumented women than in the general parturient population. The results also reveal that the coverage of prenatal screenings amongst undocumented pregnant women was inadequate.

Delayed onset of prenatal care may adversely affect the obstetric outcome and the early detection of possible obstetric complications. In addition, screening of infections, the detection, and treatment of infectious disease may be delayed. This could lead to an increased risk of mother-to-child transmissions. Consequently, undocumented women should be guaranteed extensive, equal, and timely access to prenatal care to ensure a safe and positive pregnancy outcome.

## Data Availability

The data on undocumented women analyzed in the study is available from the corresponding author on reasonable request. The data on perinatal statistics of parturients, deliveries, and newborns in 2018 that support the findings of the study is publicly available from the Finnish Institute for Health and Welfare at http://www.julkari.fi/bitstream/handle/10024/138998/Tr49_19.pdf?sequence=5&isAllowed=y. The data on infectious diseases of all pregnant women in 2017 that support the findings of the study was obtained from the Finnish Institute for Health and Welfare. The data is not publicly available.

## Abbreviations

ECDC: European Centre for Disease Prevention and Control
ESBL: Extended spectrum beta-lactamases
GC: Global Clinic, voluntary health clinic for undocumented migrants
GBS: Group B streptococcus
GW: Gestation week, the gestational age is described as completed weeks of pregnancy
HBV: Hepatitis B virus
HCV: Hepatitis C virus
HIV: Human immunodeficiency virus
MRSA: Methicillin-resistant Staphylococcus aureus
STD: Sexually transmitted diseases
